# HYBRIDMINDS—summary and outlook of the 2023 international conference on the ethics and regulation of intelligent neuroprostheses

**DOI:** 10.3389/fnhum.2024.1489307

**Published:** 2024-10-17

**Authors:** Maria Buthut, Georg Starke, Tugba Basaran Akmazoglu, Annalisa Colucci, Mareike Vermehren, Amanda van Beinum, Christoph Bublitz, Jennifer Chandler, Marcello Ienca, Surjo R. Soekadar

**Affiliations:** ^1^Clinical Neurotechnology Laboratory, Department of Psychiatry and Neurosciences at the Charité Campus Mitte, Charité – Universitätsmedizin Berlin, Berlin, Germany; ^2^Berlin Institute of Health at Charité – Universitätsmedizin Berlin, Berlin, Germany; ^3^Faculty of Medicine, Institute for History and Ethics of Medicine, Technical University of Munich, Munich, Germany; ^4^École Polytechnique Fédérale de Lausanne, College of Humanities, Lausanne, Switzerland; ^5^Faculty of Law, University of Ottawa, Ottawa, ON, Canada; ^6^Faculty of Law, University of Hamburg, Hamburg, Germany

**Keywords:** neurotechnology, neuroprosthetics, brain-computer interface, human-computer interaction, neuroethics, neurorights, regulation

## Abstract

Neurotechnology and Artificial Intelligence (AI) have developed rapidly in recent years with an increasing number of applications and AI-enabled devices that are about to enter the market. While promising to substantially improve quality of life across various severe medical conditions, there are also concerns that the convergence of these technologies, e.g., in the form of intelligent neuroprostheses, may have undesirable consequences and compromise cognitive liberty, mental integrity, or mental privacy. Therefore, various international organizations, such as the Organization for Economic Cooperation and Development (OECD) or United Nations Educational, Scientific and Cultural Organization (UNESCO), have formed initiatives to tackle such questions and develop recommendations that mitigate risks while fostering innovation. In this context, a first international conference on the ethics and regulation of intelligent neuroprostheses was held in Berlin, Germany, in autumn 2023. The conference gathered leading experts in neuroscience, engineering, ethics, law, philosophy as well as representatives of industry, policy making and the media. Here, we summarize the highlights of the conference, underline the areas in which a broad consensus was found among participants, and provide an outlook on future challenges in development, deployment, and regulation of intelligent neuroprostheses.

## 1 Introduction

The rapid convergence of neurotechnology and artificial intelligence (AI) is transforming various fields from medical treatment to human-computer interaction. Intelligent neuroprostheses and other AI-enabled devices offer significant potential to alleviate severe medical conditions, improve the quality of life, but also enhance human capabilities (Haslacher et al., 2024; Soekadar et al., 2023). Moreover, this combination of technologies gave rise to the concept of the *hybrid mind* – a state where a biological cognitive system is seamlessly merged with an artificial cognitive system (Soekadar et al., 2021). The *hybrid mind* blurs the boundary between biological and artificial intelligence, potentially leading to significant shifts in how we perceive ourselves, our identity, agency, and consciousness. While such advances promise improved cognitive functioning and extended capabilities, they also bring forward complex ethical, legal, and societal concerns, particularly related to cognitive liberty, mental integrity, and mental privacy.

In response, organizations like the OECD and UNESCO have initiated programs to address these issues and develop recommendations that balance innovation with ethical safeguards (Ienca, 2021; Bublitz J., 2024; OECD, 2019; UNESCO, 2022).

In autumn 2023, a first international conference on the ethics and regulation of intelligent neuroprostheses was held in Berlin, Germany, under the auspices of a consortium funded by ERA-NET NEURON, a network of European funding for neuroscience research. This conference gathered leading experts from neuroscience, engineering, ethics, law, philosophy, and related fields, as well as representatives from industry, policy-making bodies, and the media. The conference was divided into three sub-sections focusing on the state of the art in neurotechnology, industry perspectives and regulatory challenges. In the following, we provide an overview over key themes and give an outlook to future developments in the field.

## 2 State of the art in neurotechnology and intelligent neuroprosthetics

### 2.1 AI in neuroprosthetics: realistic applications vs. hype

Stanisa Raspopovic, professor of neuroengineering at the Swiss Federal Institute of Technology, Zurich, Switzerland, and University of Vienna, Austria, discussed the current state and challenges of integrating AI into neuroprosthetics. He emphasized the need for technologies that directly interface with the nervous system to restore sensory and motor functions or assist in pain management (Petrini et al., 2019). Raspopovic underscored that while media hype often exaggerates AI capabilities, real advancements are occurring in areas like self-learning devices, closed-loop systems, and reinforcement learning.

These advancements have led to significant improvements, allowing a positive experience of embodiment where users feel more connected to their neuroprosthetic limbs compared to previous devices. Such technologies have demonstrated practical benefits, including enhanced mobility, reduced phantom limb pain, and increased confidence (Rognini et al., 2019). These findings suggest that future research should focus on refining these biomimetic interfaces to further optimize user experience and functionality.

### 2.2 Adaptive brain stimulation and machine learning approaches

Patricia Krause, senior neurologist at Charité – Universitätsmedizin Berlin, Germany, discussed the use of deep brain stimulation (DBS) in treating Parkinson’s disease (PD), focusing particularly on recent advancements and challenges in closed-loop stimulation. She first highlighted the historical milestones in PD treatment, such as the introduction of levodopa and ablative surgeries, while emphasizing the transformative impact of DBS (Purner et al., 2023). The key PD symptoms targeted by DBS include bradykinesia, increased muscle tone, tremor, and postural instability. Krause detailed the DBS procedure, which involves implanting electrodes in specific brain regions to modulate neural activity. She stressed the importance of thorough preoperative evaluation for accurate patient selection (Barbosa et al., 2024; Pollak, 2013). Additionally, she described intraoperative and postoperative testing to ensure precise electrode placement, alongside the role of advanced imaging and algorithmic tools in enhancing treatment outcome (Haliasos et al., 2024; Huang et al., 2024; Liu et al., 2024).

One promising development is adaptive DBS, which adjusts stimulation levels based on real-time brain activity (Oehrn et al., 2024; Wilkins et al., 2024). This approach could reduce battery consumption and minimize side effects, though it still faces technical hurdles, such as signal artifacts. Krause also underscored the variability in patients’ brain signals and the impact of non-motor symptoms and emotional factors on treatment efficacy (Ledda et al., 2024). She concluded by highlighting the need for further research and development to bring closed-loop DBS into routine clinical practice. This includes refining evidence-based treatment protocols, improving artifact mitigation techniques, and delivering precise, personalized stimulation tailored to individual patients’ needs.

Julian Neumann, a clinician scientist at the same institution, highlighted the importance of understanding the effects of brain stimulation on neural activity and behavior (Horn et al., 2019). Besides concerns with hastened proliferation of DBS across psychiatric disorders without sufficient understanding of its long-term impacts beyond symptom suppression (Bublitz et al., 2023), potential risks to cognitive liberty and mental privacy were discussed, especially in cross-patient data models used to predict brain states. Neumann suggested that privacy risks could be reduced by leveraging generalized models instead of individualized data. Nonetheless, the ethical and legal challenges of adaptive neurotechnologies remain a key area for future debate.

Niels Birbaumer, pioneer in non-invasive BCI and professor emeritus in medical psychology at the University of Tübingen, Germany, provided an overview of the current state of brain-computer interface (BCI) technology, particularly focusing on its application in communication for individuals with complete paralysis, such as amyotrophy lateral sclerosis (ALS) patients (Chaudary et al., 2022), but also reporting on patients with epilepsy (Romanelli et al., 2019; Pirasteh et al., 2024). Both non-invasive and invasive BCIs were discussed (Birbaumer et al., 2014), with the invasive approach being more successful in enabling communication in completely paralyzed patients, e.g., in completely locked-in state (CLIS). He outlined various challenges in the successful establishment of BCI, such as the speed and strength of the feedback signal. He discussed the ethical implications of BCI technology, particularly for healthy individuals, and argued against using invasive BCIs in healthy people due to the limited understanding of their long-term effects. Instead, he supported restricting their use to patients with specific medical conditions. A critical risk with neurotechnologies that provide super-human abilities with an undeniable effect on the reward system would come with the risk of “Oblomovization,” a process or state of lethargy, inertia, and excessive passivity in life, often linked to the inability to take decisive action or engage meaningfully with the world (in reference to Goncharov, 1859 novel Oblomov).

### 2.3 Translating neurotechnology research into practical applications

Volker Hömberg, president of the World Federation of Neurorehabilitation (WFNR)^1^ emphasized the growing global need for multimodal neurorehabilitation (Owolabi et al., 2023). He emphasized the potential of digital tools allowing for individualized and adaptive procedures for people who have only limited access to health care and, above all, medical personnel. However, this would require the provision and accessibility of such digital tools that have so far been limited to the research domain.

Gerwin Schalk, a pioneer in BCI research (Schalk et al., 2004) working at the Frontier Lab for Applied Neurotechnology, Tianqiao and Chrissy Chen Institute, Shanghai, China, addressed the challenges of translating neurotechnology research into real-world applications. While many neurotechnology demonstrations are compelling, Schalk argued that most demonstrations fail to be scaled up to mass application. He underlined that the focus should be rather on cost-effective and commercially viable applications with a real impact on quality of life (Schalk et al., 2024) than impressive demonstrations in controlled environments that are commercially not viable and cannot be generalized. He noted that the competitive landscape of research and development underscores the importance of streamlined regulatory pathways to foster innovation.

The high cost and long timelines for developing both non-invasive and invasive neurotechnologies further complicate the transition from research to practice. Schalk emphasized that regulatory frameworks need to be adaptive enough to accommodate emerging technologies while ensuring safety and efficacy. Such frameworks should also support large-scale international collaboration to avoid regulatory fragmentation. The commercialization of neurotechnology and AI-enabled devices presents its own set of challenges, from regulatory hurdles to market competition. Industry leaders at the conference strongly agreed with this view, discussing their experiences in bringing neurotechnological products to market, particularly in Europe.

Surjo R. Soekadar, Einstein Professor of Clinical Neurotechnology and Head of the Research Division “Translation and Neurotechnology” at the Charité – Universitätsmedizin Berlin, Germany, underlined that non-invasive BCI technology is ready to be broadly applied in neurorehabilitation (Colucci et al., 2022). He pointed out that there is now ample evidence for its clinical effectiveness (Cervera et al., 2018; Yang et al., 2021), but integration of these innovative tools often complicated by regulatory challenges, lack of standardized protocols and mechanisms of reimbursement, but also insufficient training among healthcare professionals (Angerhöfer et al., 2021). Additionally, Soekadar highlighted the need for more streamlined communication between researchers, clinicians, and policymakers to ensure smooth implementation. He emphasized that while the technology is ready, scaling it for widespread clinical use requires addressing these barriers, improving accessibility, and ensuring the infrastructure supports long-term patient care.

Sumner Norman (CEO and co-founder of Forest Neurotech, USA) presented a new, non-invasive BCI approach based on focused ultrasound (Norman et al., 2021) that could potentially do both, record and stimulate neuronal activity, even in deeper areas of the brain (Rabut et al., 2024; Griggs et al., 2024). To achieve high levels of precision, a cranial window, i.e., partial removal of bone tissue, is necessary, however. While less invasive than intracranial recordings or DBS, this invasiveness limits scalability. It was noted that such bidirectional BCIs could advance neuroscientific research (Nasr et al., 2022) while opening up possibilities for numerous new and unexplored applications (Haslacher et al., 2021).

Thorsten Zander (CEO, Zander Labs, Delft, Netherlands, and professor at BTU Cottbus) highlighted the transformative potential of passive BCIs and neuroadaptive technologies beyond the medical domain (Gallegos Ayala et al., 2023; Zander et al., 2016; Klaproth et al., 2020), including personalized music recommendations and adaptive systems for elderly care. Zander advocated for the development of ethical guidelines that balance innovation with protections against misuse (Krol et al., 2020). A key concern was the potential misuse of brain data, such as for targeted advertising or surveillance. The discussion strongly emphasized the need for ethical frameworks that involve both society and researchers in shaping the future of neuroadaptive technologies.

Frank Zanow (CEO, eemagine GmbH, Berlin, Germany) shared his journey from software development to hardware innovation and regulatory navigation (Fiedler et al., 2022; Gratkowski et al., 2006). His company’s focus on high-density EEG applications for epilepsy, dementia detection, and stroke triage highlighted the potential of neurotechnology in clinical settings. However, he also emphasized the need for harmonized regulatory procedures across different countries and federal states to reduce market entry barriers.

Klaus Schellhorn (CEO, NeuroConn GmbH, Ilmenau, Germany) echoed these sentiments, pointing out the difficulties posed by the Medical Device Regulation (MDR) in Europe. He argued that the complex bureaucratic requirements disproportionately impact smaller companies, stifling innovation and limiting treatment options for conditions like epilepsy and depression (Antal et al., 2024).

Patrick Britz (CEO, NIRx GmbH, Berlin, Germany) expressed concerns about AI’s potential for misuse, contrasting medical and research applications citing examples of AI’s capabilities in predicting personal information and political leanings from minimal data (Connolly et al., 2019). Despite risks, he advocated for advancing AI in the context of neurotechnologies with caution. Regarding regulatory aspects, he emphasized distinctions between medical and research device regulations, arguing for streamlined rules to accelerate innovation. He stressed the transformative potential of affordable research tools in mental health and other fields, currently hindered by regulatory hurdles. In conclusion, he warned of missed opportunities and preventable harm if research is slowed, urging balanced regulation to foster progress while mitigating risks.

The European regulatory environment was also a key focus for Martin Schüttler (CorTec Neuro GmbH, Freiburg, Germany) who discussed his company’s efforts to bring closed-loop brain implants to first-in-human studies. Despite significant funding support in Europe, he described how regulatory challenges have forced CorTec to pursue feasibility studies in the United State. Schüttler’s experience highlights the need for more supportive regulatory environments in Europe to prevent brain drain and ensure that technological innovation supported by European public funding remains accessible to the European citizens.

In the general discussion on the perspectives from clinical research and industry, it was pointed out that the European MDR, in effect since May 2021 with transition periods until 2028, continues to present significant implementation challenges for the NeuroTech industry. Increased regulatory requirements have led to higher costs, bureaucracy, innovation loss, and longer approval processes. While recent changes to MDR in March 2023 temporarily alleviated some issues, overall, the discussion reflected that there is high demand for further adjustments. Suggestions to reform the current MDR included faster approval for ground-breaking products, reduced bureaucracy, and extending current transition periods for the different medical device classes.

The discussion also highlighted that regulatory changes do not always enhance clarity; in fact, they can often introduce new uncertainties, especially in emerging markets that are highly sensitive to such shifts. For example, BCIs do not fall under a single category within the MDR and require a case-by-case assessment based on their specific functions and intended use (Steindl, 2024). This creates challenges in determining the appropriate regulatory pathway, especially when BCIs combine hardware, software, and AI components that span different regulatory categories. The interplay between various regulatory frameworks—such as MDR, the General Data Protection Regulation (GDPR), and the now effective AI Act—demands a nuanced and comprehensive understanding, particularly in the context of neurotechnology (Soekadar et al., 2023). This complexity underscores the need for careful consideration to avoid creating additional uncertainties.

## 3 Ethical and legal dimensions of intelligent neuroprostheses

### 3.1 Philosophical, Ethical, and societal perspectives on AI and neurotechnology: debating cognitive enhancement, regulation, and public trust

Michael Pauen, professor of philosophy at the Berlin School of Mind and Brain, Germany, sketched the implications of using AI and neural implants to substitute or enhance cognitive functions from a philosophical angle. Based on the hypothesis that phenomenal and functional states are inextricably intertwined (Pauen, 2015), he argued that the substitution of cognitive functions itself should not be deemed problematic as long as the implant serves to achieve functional equivalence with the original brain processes. At the same time, he acknowledged that achieving functional equivalence remains highly difficult, given our severe lack of functional knowledge about individual brains and the complexity of cognitive abilities. Having further explored implications of neurotechnology for personal identity, free will, and responsibility, Pauen concluded by cautioning against making ambitious predictions about future capabilities of AI and neural implants.

Kostas Kostarelos, professor and chair of nanomedicine at the University of Manchester, UK, addressed the challenges and paradoxes surrounding the use of nanotechnology, particularly graphene, in neurotechnology and medical applications. He highlighted the ‘Hollywoodization’ of nanotechnology, with its portrayal in movies and media oscillating between hype and fear, and showcased the potential for recording and stimulating brain activity of graphene-based neural interfaces (Viana et al., 2024). Pointing to challenges of clinical translation and commercialization, he drew on Yuval Harari’s Homo Deus (Harari, 2016) to raise concerns about using medical applications as steppingstones for human augmentation beyond therapeutic purposes.

Gerben Meynen, a professor of forensic psychiatry at the University of Utrecht, Netherlands, explored the intersection of AI and neurotechnology within criminal law. He discussed the potential of neurobiological data to improve risk assessment and intervention strategies in forensic settings. While such data could enhance predictive accuracy for recidivism when combined with traditional factors, Meynen stressed ethical concerns surrounding coercion and valid consent, and pointed to legal obstacles advocating for a biopsychosocial model that considers social and psychological factors alongside neurobiological data (Tortora et al., 2020; Starke et al., 2023). He also highlighted the importance of securing societal and political support for rehabilitation programs that align with European human rights standards.

Pim Haselager, professor for societal implications of artificial intelligence at Radboud University Nijmegen, Netherlands, emphasized the importance of effective communication and trust-building with the general public, policymakers, and media regarding AI and neurotechnologies (Starke et al., 2022). Replying directly to earlier presentations, he stressed challenges posed by funding systems, publication biases, responsible commercialization and public perceptions influenced by hype, fear, and past controversies. Using a powerful analogy from Dutch flood control, Haselager suggested that rather than stopping emerging technologies, there is need to channel them through negotiations and finding common ground solutions.

Fred Gilbert, professor for philosophy at the University of Tasmania, Australia, discussed the potential adverse effects and personality changes associated with neurotechnologies such as DBS. Drawing on cases where patients experienced changes in emotions, impulse control, and sense of self after DBS treatment (Gilbert et al., 2017), he pointed to a potential “burden of normality,” i.e., patients’ difficulty of adjusting to being symptom-free, and to the importance of patients’ control over a device and how this impacts their experience. Turning to a case of involuntary explanation of a neurotech device that was experiences as a psychological trauma (Bublitz and Gilbert, 2023), he suggested that neurotechnology may create instances of existential dependency.

Building a bridge from ethics to regulation, Marcello Ienca from the Technical University of Munich, Germany, highlighted ethical, legal, and social implications of incorporating AI into neural interfaces (Ienca and Ignatiadis, 2020; Valeriani et al., 2022). Stressing how AI can enhance various aspects of neurotechnology for the benefit of its users, he also pointed to potential risks and ethical challenges associated with AI-enabled neurotechnology, such as dual-use for military applications, privacy infringements related to brain data, violations of mental integrity through unauthorized modifications, and ensuing challenges to personal identity. Ongoing policy work by various international organizations including the OECD, the UN, UNESCO, and the Council of Europe as well as self-regulatory efforts by research organizations such as the International Brain Initiative and the IEEE Standards Association all contribute to interdisciplinary efforts aiming for responsible innovation that does not simply restrict technology use but maximizes the benefits of technology for humanity at large.

### 3.2 Regulatory challenges and the path forward

Jennifer Chandler, professor of law at the University of Ottawa, Canada, showed how recent calls for “neurorights” reflect fundamental concerns about human interests raised by neurotechnology (Akmazoglu and Chandler, 2021). She reflected on these interests along six dimensions, differentiating physical, phenomenological, survival, functional, social, and informational interests, showing how neurotechnology could impact each of them. Given that legal systems are designed to avoid conflicts by clarifying protected interests, rights, and duties of individuals, she called for further research into the impact of neurotechnology on its users as a necessary condition for shaping appropriate legal frameworks.

Further spelling out legal concerns, Christoph Bublitz, a legal scholar from the University of Hamburg, Germany, explored four boundary cases to delineate the legal landscape regarding neurotechnology. These cases included control over bodily and mental functioning of patients using deep brain stimulation (DBS) for mood alteration, coercive mental interventions in psychiatry, the potential of ‘mind reading’ through neuroimaging and brain data decoding as well as neurotechnology-enabled workplace monitoring. Bublitz suggested potential legal frameworks, such as recognizing a ‘right to mental self-determination’ and updating data protection laws to regulate neural data processing (Bublitz and Merkel, 2014; Bublitz C., 2024).

Laura Kreiling, policy analyst at the OECD, presented the OECD’s work on responsible innovation in the context of AI and neurotechnology. Reflecting the organization’s approach to anticipatory governance of emerging technologies, her presentation focused on the OECD AI Principles and the OECD Recommendation on Responsible Innovation in Neurotechnology ado both adopted in 2019 (OECD, 2019; OECD, 2024). Drawing on examples from Germany, France, Japan, and Canada, she highlighted national implementation efforts of the guidelines, and pointed to the development of related toolkits for policymakers, industry, and academia that can support responsible innovation in the field of neurotechnology.

## 4 A balanced approach: safeguarding innovation while protecting rights

The discussions at the conference converged on the need for a balanced approach to regulation that fosters innovation while protecting fundamental human rights. Key points include:

**Focus on Medical Applications:** Development and application of intelligent neuroprostheses should focus on medical needs. Use of bidirectional BCI technologies beyond the medical domain should be restricted. Implantation of bidirectional BCIs in healthy populations should be banned as long as there is no proof for their safety and impact on long-term well-being, cognitive liberty and mental integrity.

**Preventing Overreliance on Neurotechnology:** The risk of becoming too dependent on neurotechnology for everyday functions, a phenomenon dubbed “oblomovization,” should be mitigated through public awareness and ethical oversight.

**Privacy and Mental Self-Determination:** Regulations must ensure that neural data is protected, and individuals maintain control over their cognitive functions. This includes preventing the abuse of neurotechnology by authorities or private entities. Application of intelligent neuroprostheses (particularly, bidirectional brain-computer interfaces) should be flanked by long-term studies that investigate the impact of technology use on well-being, cognitive autonomy, and personal identity. Safeguards should be in place to protect against unauthorized access and potential manipulation of neural data. Additionally, ethical frameworks should be established to address concerns related to consent, privacy, and the potential for socio-economic disparities arising from the unequal access to or misuse of neurotechnological advancements.

**Democratization and Accessibility:** Neurotechnologies should be made accessible to everyone in need of such technology and avoid the concentration of these innovations in the hands of a few large corporations. Regulatory frameworks should be designed to support smaller companies and ensure fair competition.

**Transparency and Public Engagement:** To gain societal acceptance, transparency regarding the capabilities and limitations of these technologies is crucial. Public engagement and education should be prioritized to build trust and mitigate fears.

In conclusion, the integration of AI and neurotechnology offers unprecedented opportunities for improving human health and well-being. However, these advancements come with significant ethical, legal, and societal challenges that must be carefully navigated. The discussions at the Berlin conference underscore the importance of interdisciplinary collaboration and proactive regulation in shaping a future where neurotechnological innovations can benefit humanity while safeguarding essential rights and values.

## 5 Future directions and challenges

### 5.1 Ethical governance and international cooperation

The integration of AI with neuroprosthetics and other neurotechnologies presents complex regulatory challenges that require international cooperation. A key consensus among conference participants was the need for ethical governance frameworks that are both flexible and adaptive. These frameworks should prioritize transparency, inclusivity, and accountability while fostering innovation. Participants also emphasized the importance of interdisciplinary collaboration and public engagement to ensure that societal values and human rights are adequately reflected in the development and regulation of neurotechnologies. The future of neurotechnology depends not only on technological advancements but also on our ability to navigate the ethical, legal, and social implications.

In November 2023, UNESCO Director General Audrey Azoulay appointed a 24-member Ad Hoc Expert Group (AHEG) to draft a global framework addressing the ethical challenges of neurotechnology. The initiative, mandated by UNESCO’s 194 Member States, aims to develop shared principles and actionable policies to ensure neurotechnology’s ethical use worldwide. Set for potential adoption in 2025, the recommendation seeks to balance rapid advancements with the protection of human rights and dignity, under the leadership of UNESCO’s Social and Human Science sector.

## 6 Conclusion

The convergence of neurotechnology and AI holds immense promise for improving human capabilities and addressing severe medical conditions. However, the ethical, legal, and societal challenges associated with these technologies cannot be overlooked. As highlighted during the 2023 Berlin conference, achieving a balance between innovation and ethical governance is essential for ensuring that the benefits of neurotechnology are realized while minimizing risks. International collaboration, inclusive policy-making, and robust regulatory frameworks will be key to navigating the uncertainties of this rapidly evolving field.

## Data Availability

The original contributions presented in this study are included in this article/supplementary material, further inquiries can be directed to the corresponding author.
